# Physical Model Experiments on Failure Mechanism on Slopes of Weathered Basalt Soils during Heavy Rainfall Events

**DOI:** 10.3390/ma16020832

**Published:** 2023-01-15

**Authors:** Qingzhao Zhang, Zejun Luo, Ying Chen, Zhen Wang

**Affiliations:** 1 Department of Geotechnical Engineering, Tongji University, Shanghai 200092, China; 2 School of Business Administration, Shanghai Lixin University of Accounting and Finance, Shanghai 201620, China; 3 School of Mechanical Engineering, Nanjing University of Science and Technology, Nanjing 210094, China

**Keywords:** basalt platform, rainfall intensity, weak interlayer, landslide

## Abstract

Basalt platforms are widely distributed in many areas of China, where landslides occur frequently. It is well recognized that landslide hazards seriously threaten engineering constructions and property safety. It is, therefore, of great significance to understand deformation and failure behaviors and their mechanisms in basalt slopes to reduce the loss caused by landslides. In this work, the Pengshan Landslide in Zhejiang Province is taken as a prototype and slope model tests are carried out. During the tests, real-time monitoring of pore pressure, earth pressure and slope deformation is conducted. Based on the experimental data, the influence of rainfall intensity and the thickness of a weak interlayer on the slope stability are obtained. It is demonstrated that the rainfall and weak interlayer are the most important factors causing the slope instability of a basalt platform. Furthermore, damage from a basalt platform slope usually starts from local failure, and the slope foot is the most likely sliding part. Moreover, when the rainfall intensity is doubled, the initial deformation time of the slope is reduced by about half and the final failure time is advanced by one-third. In addition, when the thickness of the weak interlayer is doubled, the initial deformation time of slope is shortened by about half and the final failure time is advanced by one-quarter.

## 1. Introduction

**Figure 1:**
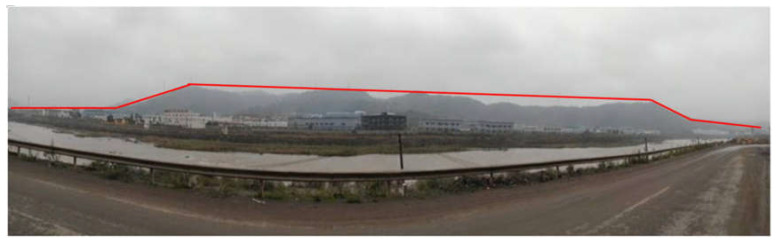
Basalt platform.

Basic or ultrabasic magma in the mantle rises along deep and large faults, sprays to the surface and then cools down and solidifies in low-lying areas. After geotectonic movement and flow erosion cutting, “topographic inversion” occurs, namely basalt platform, forming flat and steep platform-like landforms at different elevations, as shown in Figure 1. The upper part of the margin of the basalt platform is mainly composed of colluvial and slope clayey soil containing gravel and boulders, which can be characterized by low strength, while the lower part is composed of diatomite, clayey soil and other fluvial and lacustrine deposits, which can be characterized with softening in the presence of water. In addition, the weathered rock surface often forms a catchment zone and partly confined aquifer. All the above characteristics provide favorable conditions for the occurrence of basalt platform landslides. According to statistics, 267 landslide sites have been found in Shengzhou City, Xinchang County and Ninghai County, China, where basalt platforms are widely distributed. Among them, 104 landslides are basalt platform landslides, accounting for 39% of the total landslide hazards [1,2,3]. With the development of a social and economic foundation, a large number of landslides will be encountered in the construction of hydraulic, mining and transportation projects. Landslide disasters will seriously threaten the safety of construction projects and people’s lives. For example, in 1983, the landslide volume of Sale Mountain landslide in Gansu Province reached 31 million cubic meters, destroying three villages and causing 237 deaths in 30 s [4,5]. In addition, the cost of landslide control occupies a considerable proportion in the construction of the project. Taking the construction of Shangyu-Sanmen Expressway as an example, there were more than ten landslides in the basalt platform area of the construction section, and the largest one, No. 6 landslide, cost nearly CNY 100 million for its treatment [6]. It can be seen that if not dealt with in time, the impact of landslides on engineering and the damage to people’s lives can be enormous. Therefore, in order to reduce the loss caused by landslides, it is of great significance to understand the failure behavior and mechanisms of basalt platform slopes.

The occurrence of any landslides is always the result of the interaction of internal and external factors. For the surrounding rocks in basalt platform landslides, the internal factors include the topographic and geomorphological conditions, stratigraphic lithology, geological structure and engineering geological conditions of the basalt platform, while the external factors include rainfall, earthquake, human engineering activities and so on. In the area of Jiangsu and Zhejiang, where basalt platforms are widely distributed, landslide hazards are mainly concentrated in May to August, which is basically consistent with the time of concentrated rainfall in this area. Based on previous studies [7,8], it can be considered that rainfall, especially long-time heavy rain, is the main inducing factor of landslides on the basalt platform. Precipitation infiltration can increase soil water content and pore water pressure and, in turn, decrease the shear strength and lead to landslides [9]. Ng et al. [10] analyzed the influence of different rainfall duration and intensity on the stability of an unsaturated soil slope and discussed the influence of different strata and rainfall conditions on the pore water pressure distribution of the slope. It was pointed out that initial groundwater level, rainfall intensity and rainfall duration are the main factors affecting the stability of unsaturated soil slope. Through large-scale on-site experiments of artificial rainfall landslides, Hu et al. [11] discussed the effects of rainfall infiltration conditions, soil structure characteristics, rainfall capacity, rainfall intensity and other factors on rainfall-induced landslides. Zuo et al. [12] conducted model tests on three kinds of accumulated soil samples, studied the seepage, deformation, failure and particle migration of the accumulated soil slope under rainfall conditions and discussed the influence of particle gradation on the stability of the accumulated soil slope. Subsequently, other researchers have studied the failure mechanism of shallow landslides under different rainfall conditions [13]. Many conclusions have been drawn on the influence of rainfall conditions on slope stability, but there are few discussions and analyses on the whole process of slope deformation and failure during rainfall, such as the effect of rainfall intensity on the time of initial slope deformation, the time of final failure and the failure form.

In addition to rainfall, deformation of the slope is easily controlled by a weak interlayer as well [14]. When the weak interlayer meets water, it will show obvious strength softening, which makes the slope easy to slide along the weak layer. Many scholars have studied the influence of a weak interlayer on slope stability by means of field investigation and model testing. Wang et al. [15] conducted centrifugal model tests on clay slopes with weak interlayers under rainfall conditions. It was considered that the deformation of clay slopes with weak interlayers under rainfall conditions could be divided into three stages: uniform deformation stage, staggered stage and landslide stage. The existence of weak interlayers could change rainfall infiltration distribution, thus, reducing the slope stability. Jian et al. [16] analyzed the origin of No. 1 landslide in the Loess Slope of the Three Gorges Reservoir Area and considered that the soft interlayer, which widely developed in the bedrock, is the primary factor. Through borehole analysis, Xiao et al. [17] found that the rock mass in the front of the loess slope is densely interbedded, which is liable to be muddified into a landslide sliding zone and undergoes deep creep; being affected by F3 fault cutting and secondary fold development, the rock mass is broken and a landslide occurs. Although there are many studies on weak interlayers, the effects of thickness of weak interlayers on pore water pressure, earth pressure and slope deformation under rainfall conditions still need to be studied.

The vertical joints of basalt are well developed, which is conducive to the infiltration of precipitation into the underlying weak layer and can easily form a sliding surface, leading to a landslide under a small natural slope. At present, there is little research on basalt platform soil-like slopes, and there is no systematic understanding of the influence of basalt vertical joints and a weak interlayer on slope stability. Therefore, it is of great significance to study the stability of basalt platform landslides with a weak interlayer under rainfall conditions. Taking Pengshan Landslide as a prototype, this paper establishes an experimental system that can be used to simulate the deformation and failure of a slope under rainfall conditions. The physical model tests are carried out with rainfall intensity and thickness of the weak interlayer as control variables. The pore pressure, earth pressure and deformation of the slope were monitored by a pore pressure gauge, earth pressure gauge and displacement gauge in real time. Based on the experimental phenomena and data, the influence of rainfall intensity and thickness of the weak interlayer on slope stability is analyzed, which provides a reference for the deformation and failure analysis of other basalt platform slopes.

## 2. General Situation of Prototype Landslide

Pengshan Landslide is located in the gentle slope zone on the west side of the basalt platform of 104 National Highway in Shengzhou City, Zhejiang Province, China. Through field investigations, it can be seen that the height of the mountain body is about 150 m, which is characterized by gentle peaks, steep upper slope and gentle lower slope. The landslide is located in the gentle slope section at the foot of the mountain body with an elevation between approximately 45 and 120 m, and an average slope of the natural terrain between approximately 9 and 16°, where the vegetation is dense. There are temporary gullies in the lower part of the landslide, where a large amount of water occurs after rain.

**Figure 2:**
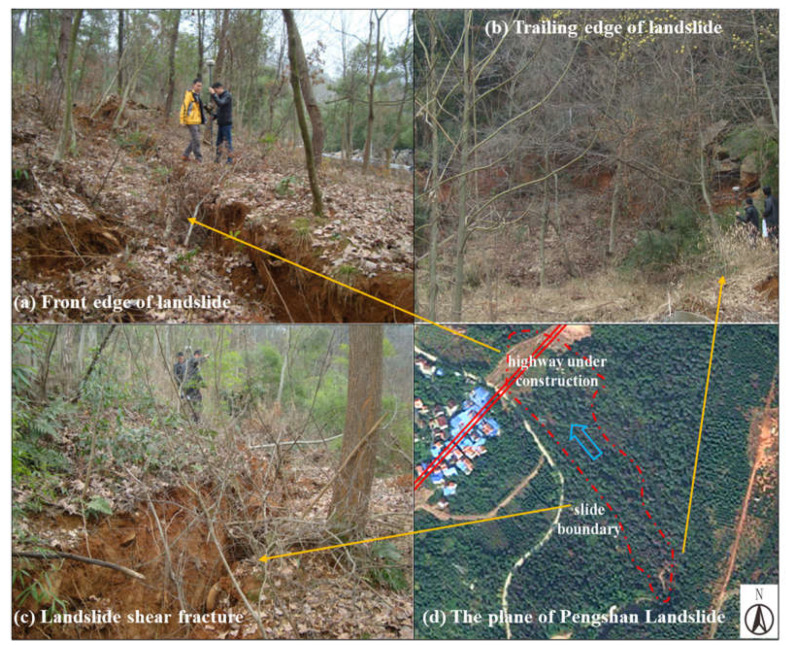
Landform characteristics of landslide: (**a**) front edge of landslide; (**b**) trailing edge of landslide; (**c**) landslide shear fracture; (**d**) the plane of Pengshan Landslide.

The Landform characteristics of Pengshan landslide are shown in Figure 2. Figure 2a–c are the front edge of the landslide, the trailing edge of the landslide and the landslide shear fracture of Pengshan Landslide. The plane of the landslide is a long strip, as shown in Figure 2d. The average width of the landslide is about 105 m, the longitudinal length is about 410 m, the area of the landslide is about 29,700 m^2^ and the thickness of the landslide body varies from 8 to 13 m. It is estimated that the volume of the landslide is about 300,000 m^3^, making it a medium-sized gravel soil landslide.

**Figure 3:**
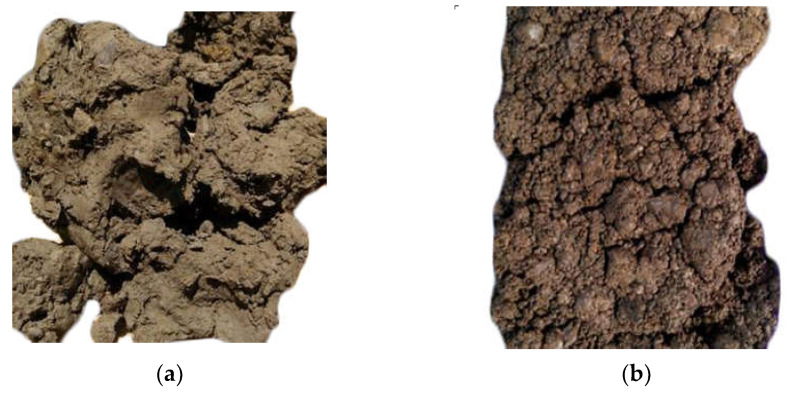
Photograph of soil sample. (**a**) clayey soil sample; (**b**) weathered basalt soil sample.

By sampling the rock and soil in the landslide area, it is found that the main landslide bodies are composed of gravel-bearing clay, gravel and tertiary basalt intercalated with thin clay. The clayey soil has a high plastic limit index, a weak permeability and its shear strength decreases rapidly because it is easy to soften in water and so the slope easily slides along this layer, as shown in Figure 3a. At the same time, the basalt is characterized by joint and fissure development. Under the influence of rainfall and other climate factors, weathering is intense. Basalt weathering has a weak–strong expansibility and other characteristics of clay. Its physical and mechanical properties are poor, indicating a great threat to slope stability as shown in Figure 3b.

In terms of climatic conditions, the annual average temperature of the landslide area is 16.4 °C, the extreme minimum temperature is −10.1 °C, the extreme maximum temperature is 40 °C, the annual average relative humidity is 82% and the annual average precipitation is 1304.8 mm. However, the distribution of rainfall is uneven, mostly concentrated in the spring rain season, rainy season in March–June and typhoon season in August–September, with annual evaporation of 800–1000 mm. There is no long-term stable surface water body in the landslide area, yet there is a large temporary flow in the low depression on the left side of the landslide body after rain. The flow rate varies from 30 to 150 tons per day.

Through the investigation of the Pengshan landslide, it is found that the main causes of the landslide are as follows: Landslide area is located on the margin of the basalt platform, and unstable slopes are often distributed on the margin of the platform. From the borehole data, it can be concluded that the clay interlayer in the basalt fragment layer has a softening phenomenon, and the slope is liable to slide along the layer. In the rainy seasons, the pore water pressure increases, the matrix suction decreases, the saturated area of the slope increases and the downward sliding force of the slope increases. In addition, the shear strength of clayey soil decreases greatly when it is softened by water, which further reduces the stability of the slope.

## 3. Physical Model Test

### 3.1. Model Design

Pengshan Mountain is characterized by a steep upper slope and gentle lower slope, and the average slope of the natural terrain is 9–16°. In addition, according to Tang’s comparative analysis of the topographic gradients of landslide geological hazards in 114 basalt platforms in Zhejiang Province [18], it is found that the landslide gradients in basalt platforms are mostly between approximately 0 and 30°, among which 10~20° is the most developed. Therefore, in the model design, the model flume is divided into three sections, each of which has a length of 2 m and a gradient of 10°, 15°and 30°, respectively. The middle section (15°) is the test section.

**Figure 4:**
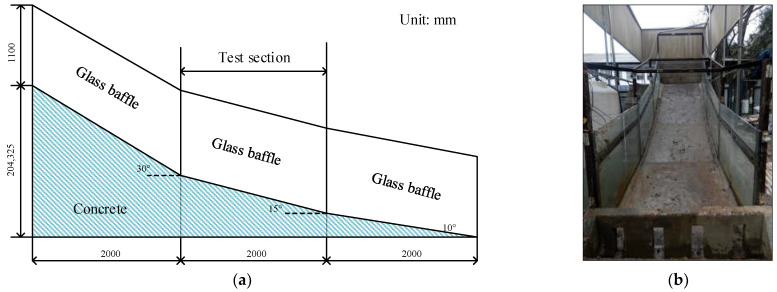
Sketch of the physical model test flume. (**a**) Model box size. (**b**) Model test flume.

The width of the model trough is 1.5 m. Groundwater depth is a critical parameter in assessing regional liquefaction potential [19,20,21]. This research is a simplified test for the Pengshan landslide. Since the main research content is rainfall intensity and weak interlayer, the influence of groundwater level is not considered. A certain thickness of concrete is poured at the bottom to simulate the bedrock and act as the waterproof boundary. Transparent glass baffles are installed on both sides of the model trough to simulate the lateral displacement limit boundary, so as to ensure that all points at the same height on the same transverse section have the same force and deformation, so that the three-dimensional seepage problem can be simplified to a two-dimensional problem in the process of data processing. The glass baffles on both sides are 1.1 m in height and are connected and fixed by angle steel and concrete at the bottom of the trough. The structure and size of the model trough are shown in Figure 4.

### 3.2. Similarity Conditions and Physical and Mechanical Parameters

According to the test conditions, the geometric similarity constant $c_{L}$ is 30, the length of the model trough is 6 m, the maximum height is 3.14 m and the width is 1.5 m. In this model test, the prototype material from the site is selected as the model. Its density similarity constant $c_{\rho }$ is 1, and consolidation is not considered in the test. According to the similarity theory [22], the time similarity constant $c_{t}\;$and rainfall intensity similarity constant $c_{q}$ of the model test can be calculated, as shown in Equations (1)–(3).

(1) $$c_{\rho }=\frac{c_{L}}{c_{t}{}^{2}}=1$$

(2) $$c_{t}=\sqrt{c_{L}}=\sqrt{30}$$

(3) $$c_{q}=c_{L}/c_{t}=\sqrt{30}$$

where $c_{\rho }$, $c_{L}$, $c_{t}$ and $c_{q}$ are density similarity constant, geometric similarity constant, time similarity constant and rainfall intensity similarity constant, respectively.

In the calculation of slope stability, the effective shear strength index is selected as the shear strength index. However, it is difficult to directly measure the effective shear strength index in the actual test. Therefore, this paper combines the total stress intensity index with the stress history to make a joint measurement, i.e., to conduct a consolidated undrained triaxial test and draw Mohr’s Circle under different confining pressure ${\;\sigma }_{3}$. The total stress intensity index of the material can be obtained from the solid line in the figure. Its value is the sum of effective stress and pore water pressure${\;u}_{f}$. However, the pore water pressure $u_{f}$ is constant during the test, so the effective shear strength index can be calculated by translating the total stress Mohr’s Circle to the left at the same time. The physical and mechanical parameters of gravel-bearing clay, basalt weathered soil and clayey soil are obtained by laboratory tests, as shown in Table 1.

### 3.3. Rainfall System

**Figure 5:**
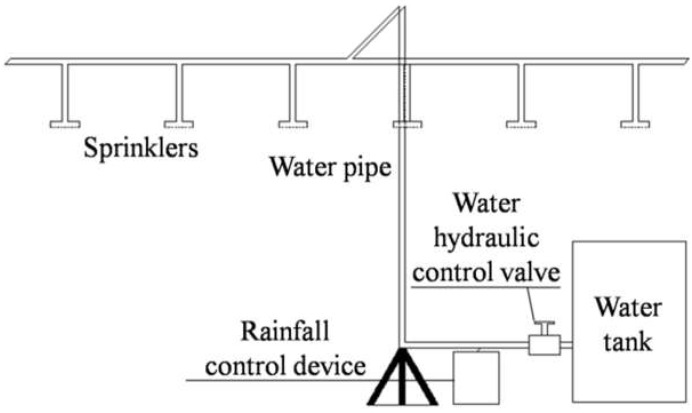
Sketch of the rainfall system.

The simulation of natural rainfall is mainly to simulate the intensity and duration of rainfall. The rainfall system designed in this experiment is composed of a QYJY-501 analog rainfall controller, 3000 L water tank, pump, rainfall water supply pipeline (support), simulated rainfall sprinkler and rainfall gauge. A structure sketch is shown in Figure 5. The rainfall intensity and duration can be set in advance. The rainfall intensity of the system can be controlled from 20 mm/h to 150 mm/h. The test shows that the maximum rainfall intensity deviation in the system is less than 3.5%, the rainfall intensity deviation coefficient is less than 3.1% and the reliability is high.

### 3.4. Test Scheme

The purpose of this experiment is to study the deformation and failure mechanism of a basalt slope with a weak layer under different rainfall conditions. Therefore, rainfall intensity and thickness of the weak layer are selected as test control variables. In the model, the slope body is paved with clay (weak interlayer), basalt weathered soil and clayey soil containing gravel obtained locally from Pengshan landslide, from bottom to top. The thickness of clayey soil, basalt weathered soil and clay (weak interlayer) in the original Pengshan landslide is about 3 m, 12 m and 3 m, respectively. The similarity constant $c_{L}$ of this model test is 30, and the thickness of each layer in the model is 10 cm, 40 cm and 10 cm, respectively. In order to further study the influence of the thickness of the weak interlayer on the stability of the slope, the conditions of no weak interlayer and 20 cm-thick weak interlayer are added, and the thickness of clayey soil with gravel and weathered soil with basalt in the upper layer is adjusted proportionally to ensure that the sum of the thickness of the soil layer on the slope is 60 cm.

For rainfall intensity, the similarity constant $c_{q}$ calculated according to the similarity ratio criterion above is $\sqrt{30}$, but according to this, the rainfall intensity of the model is only 0.8–1.9 mm/h, even if the heavy rainfall is simulated (rainfall intensity is 4.2–10.4 mm/h). The rainfall effect is very insignificant and beyond the control range of the rainfall system, so it is impossible to study slope stability under rainfall infiltration conditions. Therefore, according to the local hydrological statistics and rainfall system performance of Pengshan landslide, 30 mm/h and 60 mm/h are selected as the experimental rainfall intensity values. For each group of experiments, the total rainfall is controlled at 150 mm, that is, the rainfall duration is 5 h and 2.5 h, respectively. The test scheme is shown in Table 2.

### 3.5. Monitoring System

The monitoring system in this test includes nine pore pressure gauges, nine earth pressure gauges and two displacement gauges. The pore pressure gauge model is BWMK-0.1, the resolution ratio is 200pa/μξ, the measuring range is 0~5 MPa, the bridge resistance is 350 Ω and the calibration error is less than 0.5%. The earth pressure gauge model is BW-0.1, the resolution ratio is 200pa/μξ, the measuring range is 0~5 MPa, the bridge resistance is 350 Ω and the calibration error is less than 0.5%. The displacement gauges are measured by a percentile meter with a resolution of 0.01 mm and a measurement range of 30 mm.

**Figure 6:**
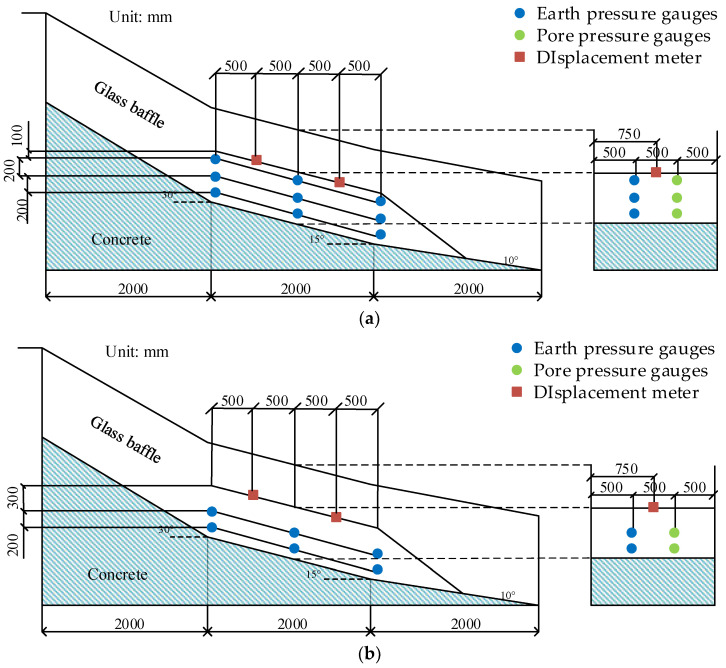
Sketch of the monitoring system. (**a**) Sensor distribution of Test 1~4. (**b**) Sensor distribution of Test 5~6.

The monitoring system is arranged as shown in Figure 6. The middle test section of the model trough is divided into three sections to observe pore pressure and soil pressure. In Test No. 1–4, three pore pressure gauges and three earth pressure gauges are placed in each section, and the buried depth is 10 cm, 30 cm and 50 cm, respectively (Figure 6a). In test No. 5–6, two pore pressure gauges and two earth pressure gauges were placed in each section, and the buried depth is 30 cm and 50 cm, respectively (Figure 6b). The pore pressure gauges and earth pressure gauges are symmetrically arranged along the axis of the model trough. Two displacement gauges measure the slope displacement by measuring the displacement changes in T-shaped steel baffles buried in the upper and lower sections of the slope. The buried depth of T-shaped steel baffles is 10 cm and the exposed part of the baffles should be perpendicular to the slope surface. By adjusting the displacement percentile position, the measuring pointer of the displacement percentile meter is perpendicular to the baffle surface.

## 4. Deformation Process and Failure Characteristics of Slope

### 4.1. Effect of Weak Layer Thickness

**Figure 7:**
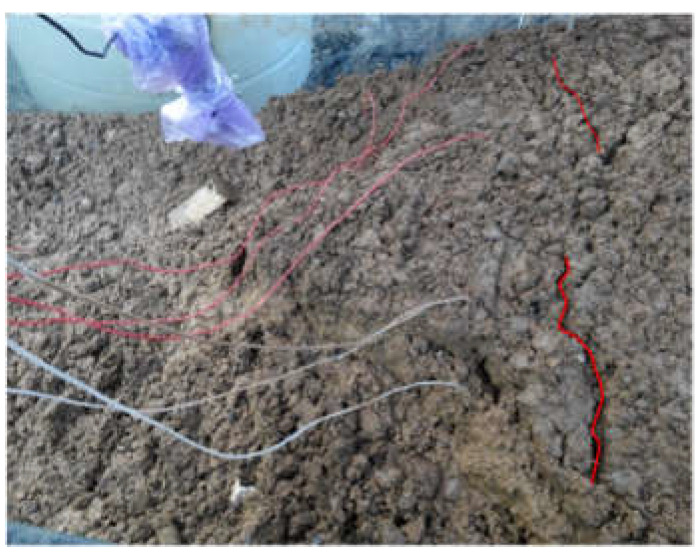
Tension cracks on the slope surface (Test 1).

**Figure 8:**
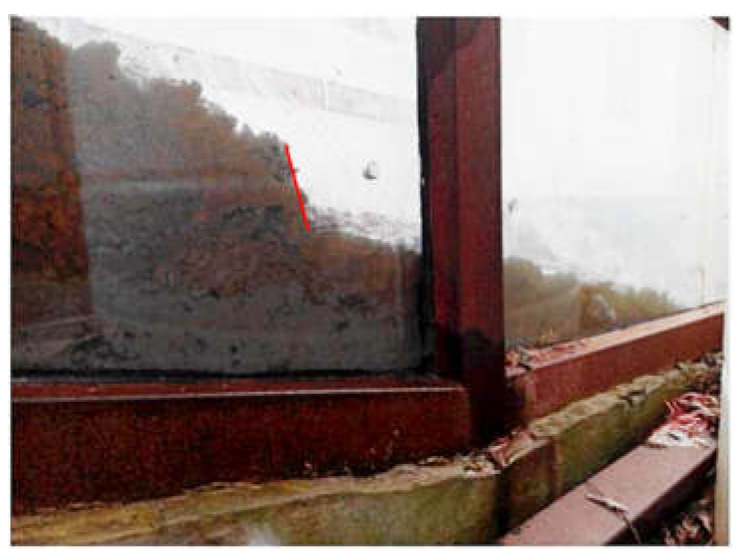
Vertical crack at the back edge of the slope (Test 1).

**Figure 9:**
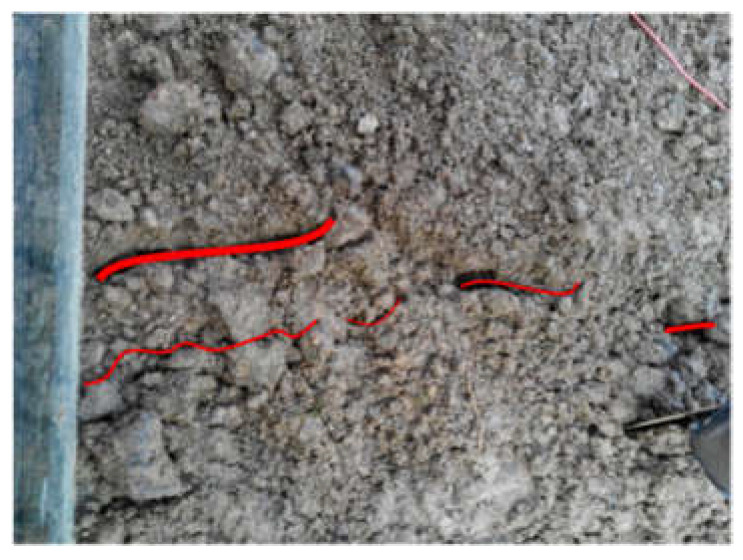
Cracks on sides of the slope (Test 3).

**Figure 10:**
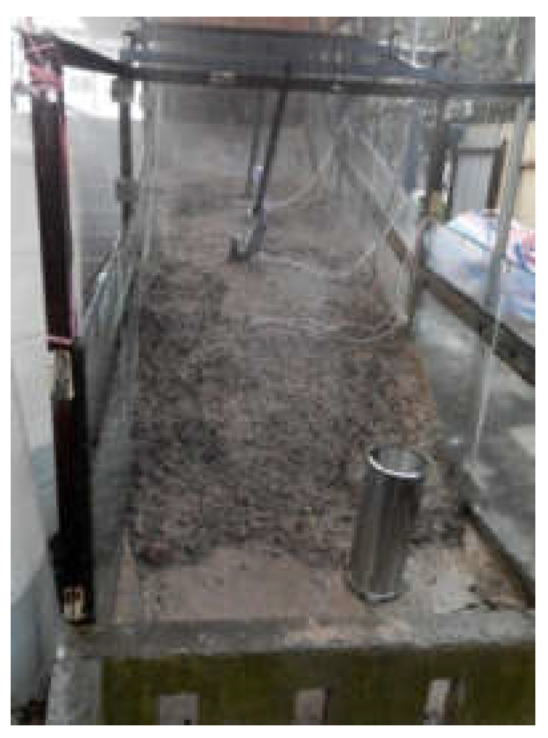
Slope morphology after test (Test 5).

Comparing the three test phenomena (Tests 1, 3 and 5) with rainfall intensity of 30 mm/h, it is found that when the test is finished, about half of the slope with a 20 cm-thick weak layer is damaged, and there are obvious tension cracks on the slope surface, as shown in Figure 7. The width of the tension crack is about 1–2 cm. The vertical crack at the back edge of the slope can also be observed from the side of the model trough, as shown in Figure 8. The slope with a 10 cm-thick weak layer has not been obviously damaged, as only slippage occurs at the foot of the slope. However, there are clear cracks on both sides of the slope, as shown in Figure 9, which shows that the stability of the slope is poor at this time. The slope without a weak layer is not destroyed, as shown in Figure 10, and a drainage channel is found at the foot of the slope. It can be inferred that the existence of the drainage channel greatly reduces the volume of the saturated area in the slope and ensures the stability of the slope. Similarly, three groups of experimental phenomena (Tests 2, 4 and 6) with 60 mm/h rainfall intensity are compared. It is found that with a decrease in the thickness of the weak layer, the stability of the slope gradually increases, from overall sliding failure to local failure to no failure.

The above experimental phenomena show that the existence of a weak interlayer in the slope has an important influence on slope stability. Compared with the weak interlayer, the permeability of basalt weathered soil is stronger, and because of the obvious development of basalt fissures, the weathered soil is obviously granular, which can easily form a flow passage in the interior of the slope. Moreover, in the process of rainfall, water can be discharged in time to stabilize the slope.

In addition, by removing the residual upper layer of gravel-bearing clay and weathered basalt soil, it can be found that the sliding distance of the weak layer is significantly shorter than that of the overlying layer (for example, in test 1, the weak interlayer only advances about 5 cm). It can be inferred that with the progress of rainfall, the slope began to slide and the main performance is that the overlying soil layer slides along the weak interlayer.

### 4.2. Effect of Rainfall Intensity

When the thickness of the weak layer is 20 cm, the experimental phenomena when the rainfall intensity is 30 mm/h and 60 mm/h (test 1 and 2) are compared, and it is found that the final failure modes of the two are similar. There are tension cracks on the slope surface and vertical cracks on the back edge of the slope. However, when the rainfall intensity is strong, the scale and number of cracks increase and there are obvious water inrush channels on the sliding surface. In addition, the beginning of sliding and the ultimate failure time of the slope are also advanced. By comparing the experimental phenomena of different rainfall intensities in the case of other weak layer thickness, we can find the same result as above, that is, rainfall intensity has little influence on the final failure form of the slope, but an increase in rainfall intensity will greatly accelerate the deformation and failure of the slope.

### 4.3. Slope Deformation

Although displacement gauges are installed on the upper and lower slopes, it is found from the monitoring results that the displacement gauges on the upper slopes are always equal to about 0. It can be concluded that no sliding occurred in the upper part of the slope during the test. This phenomenon is related to the permeability of slope material and slope structure. Because of the strong permeability of basalt weathered soil and the slope of the test section being 15 degrees, the rainwater flows downwards. Therefore, the displacement of the lower slope is significant while there is no displacement in the upper slope.

**Figure 11:**
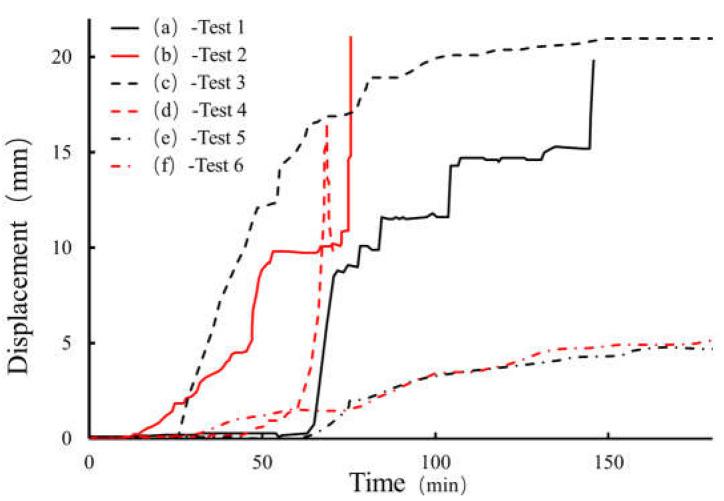
Curves of slope displacement.

Figure 11 shows the displacement curve of the lower slope with time in each group of tests. It can be seen from Figure 11a,b that when the thickness of the weak layer is 20 cm, there is a secondary sliding of the slope. Specifically, when the rainfall intensity is 30 mm/h (Figure 11a), the slope does not slip in the first 60 min. With the gradual infiltration of rainwater, the displacement of the slope increases rapidly between 60 and 90 min, and the slope begins to slip. With the continuation of the test, the deformation of the slope becomes gentle again. This is due to the sliding of the slope and the development of cracks, which drains part of the rainwater from the slope and temporarily alleviates the unstable factors. However, under the condition of continuous rainfall, after 140 min, the displacement of the slope increases sharply again, the slope slides twice and then the deformation continues to stabilize. Similarly, when the rainfall intensity is 60 mm/h (Figure 11b), the displacement does not change in the first 15 min, yet changes significantly between approximately 15 and 55 min, and the deformation of the slope tends to be gentle between approximately 55 and 80 min. After 80 min, the slope undergoes a second sharp deformation, which results in the slide of the displacement gauge and makes it impossible for subsequent measurement.

Figure 11c shows the slope displacement curve of Test 3 (the thickness of the weak layer is 10 cm and the rainfall intensity is 30 mm/h). It can be seen from the figure that the slope begins to slide in 30 min, reaches maximum displacement in 90 min and then remains stable, that is, there is not sliding failure of the slope.

Figure 11d shows the slope displacement curve of Test 4 (the thickness of the weak layer is 10 cm and the rainfall intensity is 60 mm/h). It can be seen from the figure that there is no deformation on the slope in the first 45 min and slow deformation occurs on the slope between approximately 45 and 60 min, but then the displacement of the slope increases sharply, showing that the slope is destroyed by instability, resulting in the slide of the displacement gauge.

Figure 11e,f show the slope displacement curves without a weak interlayer. It can be seen from the figure that both slopes reach the maximum displacement (about 5 mm) when rainfall stops. When rainfall stops, it can be observed that they still remain stable.

In summary, only in the case of heavy rainfall or a thick weak interlayer, obvious slope deformation and failure will occur, and the failure process happens gradually.

## 5. Analysis of Internal Monitoring Results of Slope

### 5.1. Change in Pore Water Pressure in Slopes

**Figure 12:**
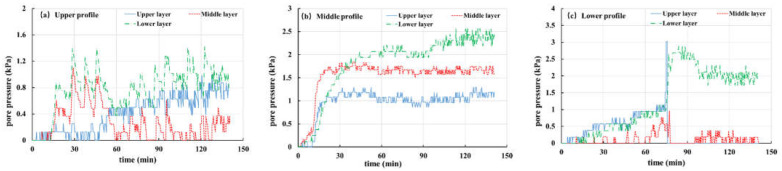
Curves of pore pressure in Test 2 (20 mm, 60 mm/h): (**a**) Upper profile; (**b**) Middle profile; (**c**) Lower profile.

**Figure 13:**
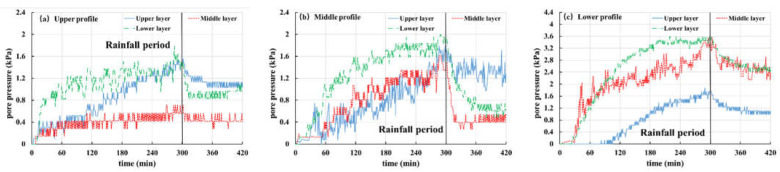
Curves of pore pressure in Test 3 (10 mm, 30 mm/h): (**a**) upper profile; (**b**) middle profile; (**c**) lower profile.

**Figure 14:**
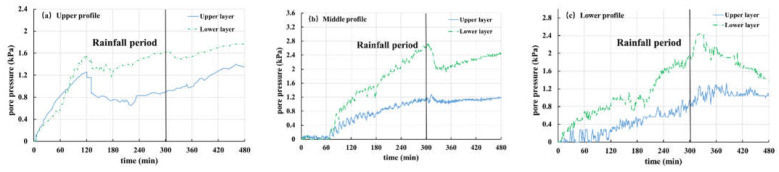
Curves of pore pressure in Test 5 (-, 30 mm/h): (**a**) upper profile; (**b**) middle profile; (**c**) lower profile.

During the experiment, the pore pressure changes in the slope are recorded by the pore pressure gauge embedded in the slope. In this paper, Tests 2, 3 and 5 are selected as examples to analyze and discuss the pore pressure, as shown in Figure 12, Figure 13 and Figure 14.

Figure 12 shows the pore pressure variation curve of Test 2 (the thickness of the weak layer is 20 cm and the rainfall intensity is 60 mm/h). The buried depth of pore pressure gauges in the upper, middle and lower layers is 10 cm, 30 cm and 50 cm, respectively. From the figure, it can be seen that the pore pressure of each site is rising, starting from 15 min, and rainwater gradually infiltrates into the slope. The pore pressure in the middle section (Figure 12a) is particularly obvious. The pore pressure remains stable until about 45 min, indicating that the interior of the slope reaches a saturated state. A significant fluctuation in pore pressure was observed in about 80 min (Figure 12b). Combining with the previous slope displacement monitoring results (Figure 11b) and experimental phenomena, it is found that the slope slides at this time, leading to the upper pore pressure gauge sliding along with the soil.

Figure 13 and Figure 14 show the pore pressure curves of slopes with a 10 cm-thick weak layer and slope without a weak layer under rainfall intensity of 30 mm/h, respectively. The variation in pore pressure at each point of the two groups is the same, i.e., during the rainfall period (the first 300 min), pore pressure gradually increases with rainfall infiltration, and the increase in pore pressure increases from the top section to the bottom section in turn. When rainfall stops, the curve shows that pore pressure is also gradually dissipating. It should be pointed out that the pore pressure value in the middle layer of Figure 13a is small and has no obvious change. This happens because the pore pressure gauge is located in the differentiated basalt soil and its permeability is strong. A flow passage is formed around the buried position of the pore pressure gauge, which is conducive to the rapid discharge of groundwater, resulting in little change in pore pressure during the whole process.

### 5.2. Change in Earth Pressure in Slopes

**Figure 15:**
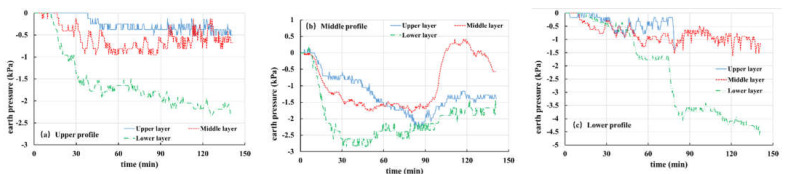
Curves of earth pressure in Test 2 (20 mm, 60 mm/h): (**a**) upper profile; (**b**) middle profile; (**c**) lower profile.

**Figure 16:**
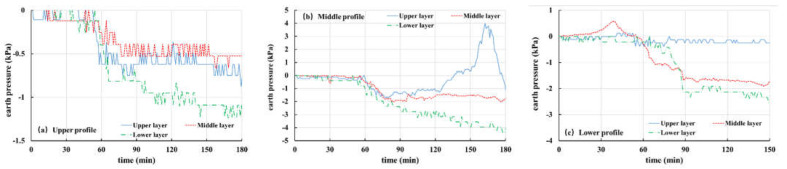
Curves of earth pressure in Test 1 (20 mm, 30 mm/h): (**a**) upper profile; (**b**) middle profile; (**c**) lower profile.

**Figure 17:**
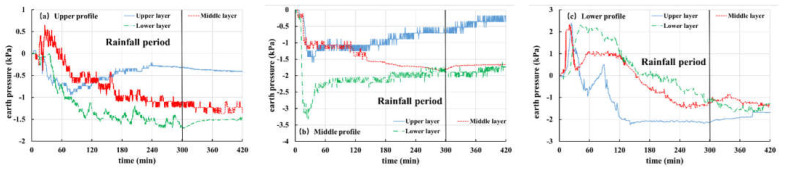
Curves of earth pressure in Test 3 (10 mm, 30 mm/h): (**a**) upper profile; (**b**) middle profile; (**c**) lower profile.

**Figure 18:**
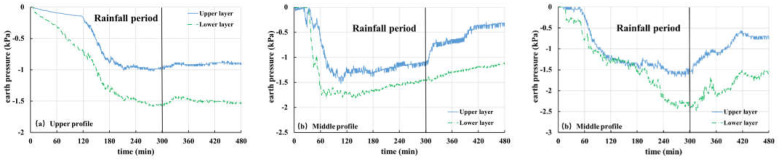
Curves of earth pressure in Test 5 (-, 30 mm/h): (**a**) upper profile; (**b**) middle profile; (**c**) lower profile.

In the present work, the overlying load of strain gauge is measured by an earth pressure gauge, which includes pore water pressure and contact pressure of soil particles. Corresponding to pore water pressure, Tests 1, 2, 3 and 5 are selected for analysis and discussion of earth pressure, as shown in Figure 15, Figure 16, Figure 17 and Figure 18.

Figure 15 shows the soil pressure variation curve from Test 2 (the thickness of the weak layer is 20 cm and the rainfall intensity is 60 mm/h). It can be seen from Figure 15a that the earth pressure of the upper section keeps decreasing and finally tends to be stable. This is because of the infiltration of rainfall, which makes the interior of the slope gradually change from unsaturated to saturated. In addition, as the burial depth of the earth pressure gauge increases gradually from top to bottom, the decrease rate of change in the earth pressure in each layer increases gradually. For the middle section (Figure 15b), the soil pressure changes rapidly in the first 45 min and is relatively stable for 45–80 min, especially the middle and lower layers of soil, which reach a saturation state. However, because of the shallow burial depth, the upper soil pressure gauge is greatly affected by rainfall infiltration, so it still shows a trend of slow decline. However, after 80 min, the three curves all have an obvious upward phenomenon, which is related to the front sliding of the slope body at this time and is caused by the drainage of water inside the slope body. Particularly, the middle-level curve rises most notably, because the middle-level earth pressure gauge is located in the weathered basalt soil layer. When the cracks develop, it is easy to form drainage channels, and when the slope slides, groundwater flows out rapidly. Similarly, it can be seen from the earth pressure curve of the lower section (Figure 15c) that the earth pressure changes obviously in 45 min. Combined with the experimental phenomena, it is found that this is caused by the local sliding of the front part of the slope. At 80 min, a sharp decline occurs again, indicating that the slope has undergone secondary sliding.

Figure 16 shows the soil pressure variation curve for Test 1 (the thickness of the weak layer is 20 cm and the rainfall intensity is 30 mm/h). The variation law of the curve is basically the same as that of Test 2. The earth pressure at each monitoring point remained stable in the first 60 min and then decreased dramatically, which is related to the sliding of the front part of the slope, and then the earth pressure at each point tends to be stable. At 160 min, the upper soil pressure curve of the middle section (Figure 16b) shows a peak value, which is different from that of the middle and lower layers. This is because the slope slides again and the upper earth pressure gauge on the middle section flips, resulting in errors in data monitoring results. In addition, at 160 min, the upper soil pressure curve of the lower section (Figure 16c) drops sharply, but it remained almost unchanged before. The reason is that the buried position of the earth pressure gauge is shallow and it is difficult for the groundwater level to reach this point. At this time, the front of the slope slides, which leads to the slide of overlying soil, thus, resulting in a sudden drop in soil pressure.

Figure 17 shows the soil pressure variation curve of Test 3 (the thickness of the weak layer is 10 cm and the rainfall intensity is 30 mm/h). The soil pressure at each point of the upper section (Figure 17a) maintains a rapid decline trend in the first 120 min and then shows a continuous and gentle decline. The soil pressure of each layer in the middle section (Figure 17b) decreases rapidly in the initial stage of the test and then it becomes more stable. Combined with the test phenomena, it is found that the soil in the front of the slope cracks and forms drainage channels at this time. With the continuous rainfall, the saturated area in the slope increases continuously and the response of the unsaturated area decreases. However, since there is limited space for groundwater level to rise and being influenced by the 15° slope of the model trough, at this time, the infiltration of rainwater is equivalent to the drainage capacity of the section, which leads to the stability of the earth pressure curve. From 30 min to 120 min, it was found that the front of the slope cracks slowly, and the crack is located just above the sensor in the lower section, which caused a strong fluctuation in soil pressure (Figure 17c). The shallower the buried position, the stronger the influence and the greater the curve fluctuation. After 120 min, the curve maintains a steady downward trend, and the rainwater received by the slope will flow through the lower section. However, the discharge capacity of the slope is limited, so it can be seen that the soil pressure of the lower section still declines after the curves of the upper and middle sections remain stable. When the rainfall stops, pore pressure dissipates and soil pressure rises correspondingly.

Figure 18 shows the soil pressure variation curve of Test 5 (there is no weak layer and the rainfall intensity is 30 mm/h). It can be seen from the graph that with the progress of rainfall, the soil pressure at each point has a downward trend. After the rainfall stops (300 min), the soil pressure rises again. It should be pointed out that the recovery of earth pressure in the upper section (Figure 18a) is not very obvious, because the upper section is far away from the front of slope deformation. In addition, the earth pressure on the middle section (Figure 18b) decreases rapidly because the difference between rainfall recharge received by the section and the discharge of the section is larger than that of other sections, resulting in a faster growth rate of groundwater level.

In general, the soil pressure in the test is affected by pore water and soil sliding. When the slope does not undergo obvious deformation, its curve variation characteristics are basically consistent with pore pressure, and when the slope undergoes large deformation, the change in soil pressure is more complex.

## 6. Discussion and Analysis

### 6.1. Relationship between Rainfall Intensity and Slope Deformation

There are two explanations for the influence of rainfall on slope stability. (1) As rainfall infiltrates the slope, the self-weight stress of the slope increases. (2) Rainfall infiltration increases the pore water pressure inside the slope and reduces the sliding resistance of the slope. According to past experience, long-duration and low-intensity rainfall can infiltrate into the deeper soil layer and easily trigger deep sliding. Short-duration and high-intensity rainfall can form transient saturation on the surface of the slope and easily cause shallow landslides. In this paper, because of the large permeability coefficient of the upper and middle layers of soil, there is no transient saturation zone in the shallow layer, and rainwater infiltrated into the interior of the slope in a relatively short time. The water storage capacity of the slope is shown in Equation (4):

(4) $$Q_{s}=Q_{i}-Q_{d}=q_{s}t-q_{d}t$$

where $Q_{s}$ is water storage, $Q_{i}$ is infiltration, $Q_{d}$ is drainage, $q_{s}$ is recharge strength, $q_{d}$ is drainage strength and t is time.

**Figure 19:**
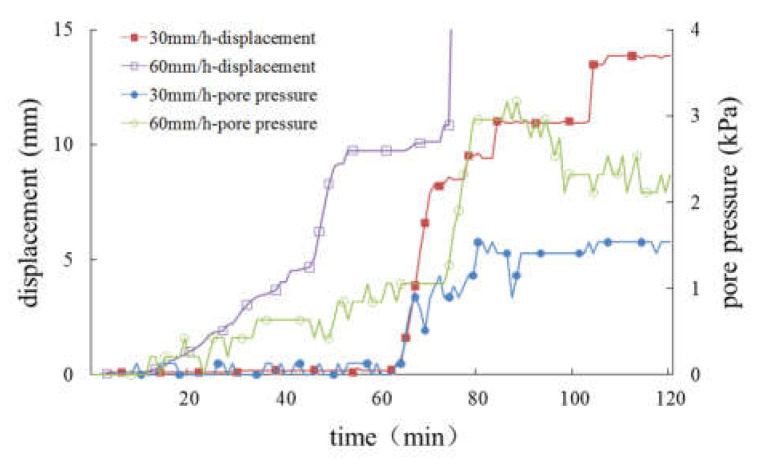
Curves of displacement and pore pressure in the slope with a weak layer of 20 cm.

**Figure 20:**
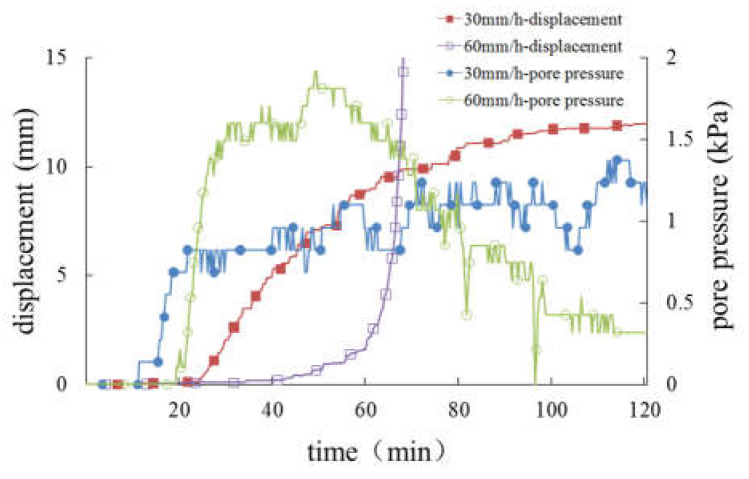
Curves of displacement and pore pressure in the slope with a weak layer of 10 cm.

**Figure 21:**
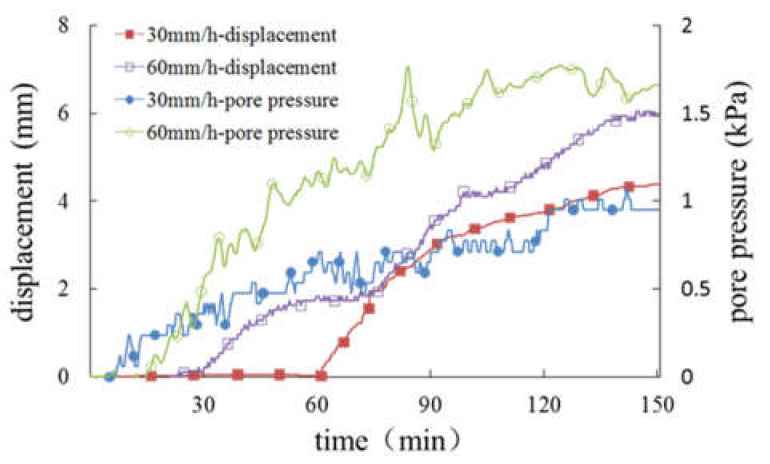
Curves of displacement and pore pressure in the slope without weak layer.

It can be seen from the above formula that high-intensity rainfall means more rain infiltration. Under the condition of constant drainage conditions, high-intensity rainfall increases the water storage capacity in the slope, which leads to an increase in pore water pressure. From the previous analysis, it is known that the pore water pressure is consistent with the change in the slope displacement. Rainfall changes the stress field of the slope by changing the pore water pressure inside the slope. The increase in pore water pressure will reduce the stability of the slope, and the decrease in stability will lead to an increase in sliding displacement, which is the fundamental reason for the synchronization of pore water pressure and slope displacement. In addition, high-intensity rainfall shows faster slope deformation. For further analysis, the effects of different rainfall intensity on pore pressure and slope displacement are given in Figure 19, Figure 20 and Figure 21.

For slopes with progressive sliding failure, the displacement and deformation curves are ladder-like, as shown in Figure 19, and the first obvious deformation displacement is defined as the start time of failure. The slope with a 20 cm-thick weak layer (Figure 19) was destroyed under two different rainfall intensities. The larger the rainfall intensity, the earlier the displacement of the slope body occurs. However, before sliding failure, the displacement of the slope surface is approximately equal. In addition, pore pressure in the slope body increases with an increase in rainfall intensity. In the slope with a 10 cm-thick weak layer (Figure 20), sliding failure occurs under the condition of rainfall intensity of 60 mm/h, while no failure occurs under the condition of 30 mm/h, and the greater the rainfall intensity, the greater the final displacement of the slope surface. For pore pressure, before the slope is destroyed, the increase in pore pressure will be faster if the rainfall intensity is strong; after the slope is destroyed, due to the adjustment of the internal structure of the soil, the situation is more complex, which may form a good drainage channel, leading to a gradual decline in pore pressure with the continuation of rainfall. It can be seen from Figure 21 that the slope without a weak interlayer was not destroyed under different rainfall intensity conditions, but the larger the rainfall intensity, the larger the displacement of the slope surface, and the earlier the start time, a similar rule of pore pressure will be present in the slope body.

According to the above analysis, it can be found that for the destroyed slope, when the rainfall intensity doubles, the initial deformation time of the slope is shortened by about half and the ultimate failure time is shortened by about one-third. In addition, the greater the thickness of the weak layer, the more obvious the phenomenon is; for the undestroyed slope, when the rainfall intensity doubles, the final displacement of the slope increases by about one-third.

### 6.2. Relation between Weak Interlayer and Slope Deformation

**Figure 22:**
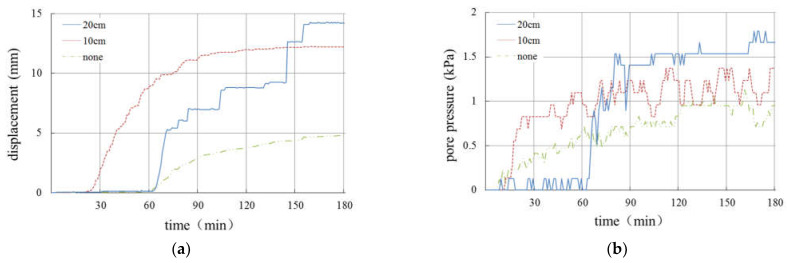
Curves of displacement and pore pressure under rainfall intensity of 30 mm/h. (**a**) Curves of displacement. (**b**) Curves of pore pressure.

**Figure 23:**
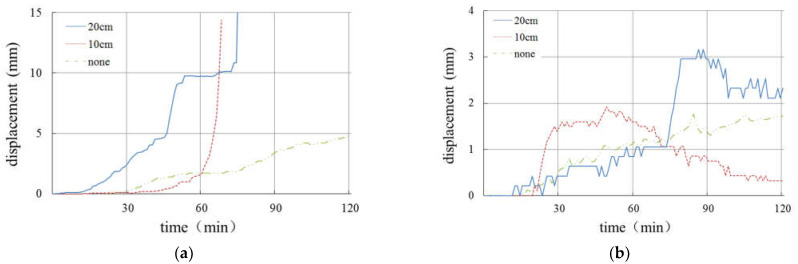
Curves of displacement and pore pressure under rainfall intensity of 60 mm/h. (**a**) Curves of displacement. (**b**) Curves of pore pressure.

Figure 22 and Figure 23 show the variation in pore pressure and slope displacement of the slopes with different thickness of the weak layer under the same rainfall conditions, so as to analyze and discuss the relationship between the weak interlayer and slope deformation.

According to Figure 22, under rainfall intensity of 30 mm/h, only the slope with a 20 cm-thick weak layer has sliding failure. The slope with a 10 cm-thick weak layer has an obvious cracking phenomenon, while the slope without a weak layer has only slight sliding on the surface. In terms of the slope displacement, the greater the thickness of the weak layer, the greater the total amount of slope deformation. Meanwhile, pore pressure in the slope increases with the increase in the thickness of the weak layer.

As can be seen from Figure 23, when the rainfall intensity is 60 mm/h, only the slope without a weak layer was not damaged, and the other two groups of slopes were damaged. With the increase in the thickness of the weak layer, the increase rate of the slope displacement increases in turn, and the displacement amount also increases. Because of the internal structure adjustment of the damaged soil body, the change in soil pressure is complex. If only the pore pressure changes before the failure is considered, it can be found that the pore pressure increases with the increase in the thickness of the weak layer.

Based on the above analysis, it can be concluded that the initial deformation time of the slope will be shortened by half if the thickness of the weak layer is doubled, and the final failure time of the slope will be advanced by about 1/4, and the increase in the deformation of the slope surface will increase sharply before the failure. For the undamaged slope, the greater the thickness of the weak layer is, the larger the deformation of the slope will be, and when the thickness of the weak layer doubles, the deformation is increased by 1/3.

### 6.3. Limitations

Due to the limitations of test conditions, concrete interface is adopted for the bedrock face in the test, which is different from the actual distribution of bedrock. It is suggested to use better materials in the future to reduce the impact of difference between contact surfaces. In addition, the slope is constant in the test, but the slope has a certain influence on the seepage and stability of soil mass. It is suggested to take the slope rate as the control variable to conduct a series of studies. Groundwater depth is a critical parameter in assessing regional liquefaction potential. Since the main research content is rainfall intensity and weak interlayer, the influence of groundwater level is not considered. In future studies, the influence of groundwater will be further considered

## 7. Conclusions

Taking Pengshan Landslide in the basalt platform area as a prototype, this paper carries out physical model tests. Through monitoring the changes in the slope displacement, pore water pressure and earth pressure in the slope during the test, the influence of rainfall intensity and thickness of the weak layer on slope stability is analyzed. Based on the test results and theoretical analysis, the following conclusions are drawn:

(1) The damage of the basalt platform slope usually starts from local failure, and the foot of the slope is the place where sliding occurs most easily; in addition, the sliding trailing edge is vertically downward and tangential, and there are many tension cracks on the slope.
(2) Rainfall is an important factor causing slope instability on the basalt platform. Rainfall changes the stress field state of the slope by changing the magnitude of pore water pressure inside the slope, which reduces the stability of the slope. Under the condition that the total amount of rainfall is the same, the greater the intensity of rainfall, the faster the growth of pore water pressure.
(3) For the slope that is eventually destroyed, the displacement change speed is similar before the slope is destroyed under different rainfall intensity. When the rainfall intensity doubles, the initial deformation time of the slope is reduced by about half and the final failure time is advanced by one-third, and the phenomenon is more obvious if the weak layer is thicker. For the undamaged slope, when the rainfall intensity doubles, the final displacement of the slope increases by about one-third.
(4) The thickness of the weak interlayer in the basalt platform slope has an important influence on the stability of the slope. For the eventually destroyed slope, when the thickness of the weak interlayer doubles, the initial deformation time of the slope is shortened by about half and the failure time of the slope is advanced by one-quarter. For the undamaged slope, when the thickness of the weak layer is doubled, the final deformation is increased by about one-third.

## Figures and Tables

**Table 1:** Material properties of the test soil.

Test Sample	$\mathrm{Bulk}\;\mathrm{Density}\;(g/cm^{3})$	$\mathrm{Saturated}\;\mathrm{Density}\;(g/cm^{3})$	Permeability Coefficient (m/s)	Natural Water Content (%)	Internal Friction Angle (°)	Cohesion (kPa)
Gravel-bearing clay	1.95	2.42	$1.53\times 10^{-5}$	20	9.5	15
Weathered basalt	1.78	2.23	$1.12\times 10^{-5}$	15	25	24
Clayey soil	1.62	2.1	$8\times 10^{-7}$	27	24	20

**Table 2:** Test scheme.

Test Number	Thickness (cm)			Rainfall Intensity (mm/h)
	Upper Layer	Middle Layer	Lower Layer	
	Gravel-Bearing Clay	Weathered Basalt	Weak Layer (Clayey Soil)	
1	8	32	20	30
2	8	32	20	60
3	10	40	10	30
4	10	40	10	60
5	12	48	0	30
6	12	48	0	60

## Data Availability

All data, models and code generated or used during the study appear in the submitted article.
